# Factors Regulating the Activity of LINE1 Retrotransposons

**DOI:** 10.3390/genes12101562

**Published:** 2021-09-30

**Authors:** Maria Sergeevna Protasova, Tatiana Vladimirovna Andreeva, Evgeny Ivanovich Rogaev

**Affiliations:** 1 Laboratory of Evolutionary Genomics, Department of Genomics and Human Genetics, Vavilov Institute of General Genetics, Russian Academy of Sciences, 119333 Moscow, Russia; andreeva@rogaevlab.ru; 2 Center for Genetics and Genetic Technologies, Faculty of Biology, Lomonosov Moscow State University, 119192 Moscow, Russia; 3 Center for Genetics and Life Science, Department of Genetics, Sirius University of Science and Technology, 354340 Sochi, Russia; 4 Department of Psychiatry, UMass Chan Medical School, 222 Maple Ave, Reed-Rose-Gordon Building, Shrewsbury, MA 01545, USA

**Keywords:** LINE-1 retrotransposons, L1 silencing, repetitive elements, regulation

## Abstract

LINE-1 (L1) is a class of autonomous mobile genetic elements that form somatic mosaicisms in various tissues of the organism. The activity of L1 retrotransposons is strictly controlled by many factors in somatic and germ cells at all stages of ontogenesis. Alteration of L1 activity was noted in a number of diseases: in neuropsychiatric and autoimmune diseases, as well as in various forms of cancer. Altered activity of L1 retrotransposons for some pathologies is associated with epigenetic changes and defects in the genes involved in their repression. This review discusses the molecular genetic mechanisms of the retrotransposition and regulation of the activity of L1 elements. The contribution of various factors controlling the expression and distribution of L1 elements in the genome occurs at all stages of the retrotransposition. The regulation of L1 elements at the transcriptional, post-transcriptional and integration into the genome stages is described in detail. Finally, this review also focuses on the evolutionary aspects of L1 accumulation and their interplay with the host regulation system.

## 1. Introduction

Dispersed DNA repeats of LINE-1 (L1) retrotransposons account for 17% of the human genome [1]. Most of L1, which includes more than 500 thousand copies, is not active in the genome as they are truncated repeats or contain mutations in the protein-coding sequence necessary for retrotransposition [2]. However, approximately 150 copies are full-length and capable of self-copying and distributing in the genome [3,4,5]. Moreover, L1 elements provoke the spread of other genetic repeats, such as Alu and SVA [6,7,8]. Insertions of L1 elements occur mainly in the non-coding regions of the genome: introns and intergenic spaces [9]. The presence of the L1 element at a certain locus can affect gene expression and even lead to the formation of alternative transcripts, which can make a significant contribution to the functions of individual cells, tissues, and the whole organism [10,11]. Regulation of L1 is a complex process in which a large number of genetic factors are involved. This review summarizes the data on molecular genetic factors involved in the regulation of L1 elements and their effect on various stages of the retrotransposition process.

## 2. L1 Structure

**Figure 1:**
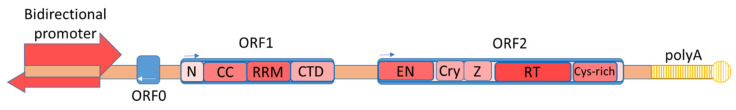
The structure of a full-length copy of L1 retrotransposon. ORF1 consists of an N-terminal domain (N), a coiled-coil domain (CCD), an RNA recognition motive (RRM), and a C-terminal domain (CTD) [18]. ORF2 consists of endonuclease (EN), retrotransposase (RT), a cryptic domain (Cry), a Z-domain (Z), and a C-terminal domain with a cysteine-rich region (Cys-rich) [24].

A full-length L1 copy is about 6 kbp. It contains a bi-directional promoter in the 5′UTR; two open reading frames, namely ORF1 and ORF2, and a 3′UTR with a polyadenylation signal (polyA) (Figure 1) [12,13,14]. ORF1 encodes a ~40 kD protein with the chaperone activity necessary to stabilize a new L1 copy [15,16,17,18]. ORF2 encodes ~150 kD proteins with the endonuclease and retrotransposase activity required for the retrotransposition process [15,19,20]. In the opposite direction, ORF0 and two antisense promoters are located in the 5′and 3′ UTR. The function of ORF0 remains poorly understood. According to some data, ORF0 is involved in the formation of chimeric proteins or enhances the mobility of L1 [13,21,22,23].

## 3. Retrotransposition Mechanism

**Figure 2:**
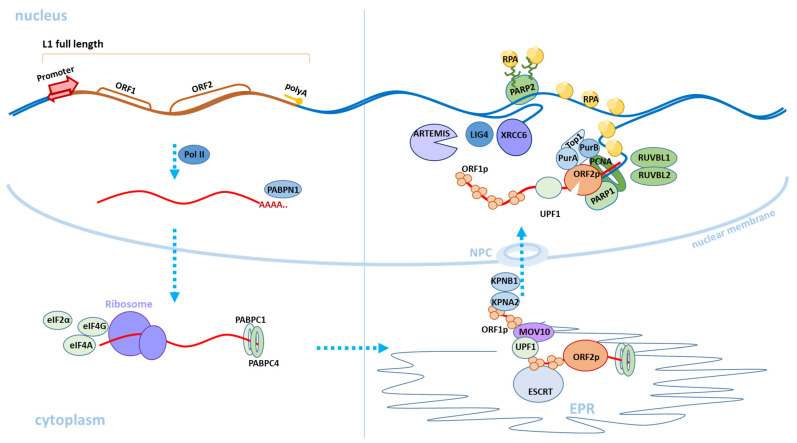
Scheme of the classical retrotransposition mechanism of L1. The transition of L1 from one stage of retrotransposition to another is indicated by blue dashed arrows. The upper left part of the figure shows the expression of a full-length copy of active L1 in the cell nucleus. The L1 RNA transcript (marked in red) is transported to the cytoplasm. The L1 ORF1p and ORF2p proteins are synthesized and the L1 RNP is formed (in the lower right part of the figure). Then, through the endoplasmic reticulum (EPR) and nuclear pore complex (NPC), L1 RNP is transported to the nucleus and L1 DNA copy formed by a reverse transcription is integrated into a new genomic locus (in the upper right part of the figure). The cellular factors involved in the retrotransposition process, which are described in this review, are also depicted.

L1 is distributed in the genome via the target-site primed reverse transcription (TPRT) mechanism (Figure 2) [20,25,26]. A new RNA copy of the L1 element is expressed from the forward chain of the L1 element due to the strong promoter in the 5′UTR [14,27]. The new copy is then polyadenylated and leaves the nucleus [28]. Translation of ORF1 and ORF2 as well as the formation of L1 ribonucleoprotein (L1 RNP) occur in the cytoplasm. L1 RNP is a bicistronic mRNA coated with ORF1 proteins and contains one or two copies of the ORF2 protein [29]. In the cytoplasm, polyadenylate-binding protein 1 (PABPC1) attaches to the polyA tail of L1; its presence is critical for the formation of L1 RNP [28]. Transportation of L1 RNP from the cytoplasm to the nucleus is accomplished using the membrane-associated endosomal sorting complex required for transportation (ESCRT) [30]. It has also recently been shown that the ORF1 protein interacts with the KPNA2 and KPNB1 karyopherins, as well as possibly other KPNA family proteins involved in nuclear protein importation through nuclear pores (nuclear pore complex) [18]. The cancer cell model showed that L1 RNP penetrates the nucleus during mitosis and the integration of the new copy of the L1 element into the genome occurs in the S phase of a cell cycle [31]. Interestingly, in different tissues, the association of the L1 retrotransposition process with certain stages of the cell cycle can be different. For example, in neuronal cell cultures, it has been shown that retrotransposition can occur in non-dividing cells [32]. In entering the nucleus and reaching the genomic DNA, the endonuclease recognizes the concensus cleavage site 5′-TTTT/AA-3′ [20,33,34,35], and creates a single-stranded DNA break with the formation of both phosphate 5′-PO4 and hydroxyl 3′-OH groups at the ends [20]. The L1 transcript is attached via the polyA tail to the region of the endonuclease recognition site and reverse transcription of the L1 RNA occurs [36,37,38]. For the classical mechanism of retrotransposition, host proteins involved in DNA repair and replication are necessary [34,39,40]. A complex of PARP1 and PARP2 proteins is formed at the single-strand DNA breaks [40]. PARP2 specifically recognizes a single-stranded DNA gap at the L1 integration site. PARP2 is activated by the poly-ADP ribosylation process (PARylation). Activated PARP2 interacts with the RPA complex, which allows for the integration of a new synthesized L1 copy into the DNA. RPA, a replicative complex of protein A (heterotrimeric protein A complex), consisting of RPA70, RPA32, and RPA14 proteins, is required to bind single-stranded DNA in eukaryotes and to protect it from the deamination of cytidine [41]. The role of PARP1 in L1 retrotransposition has not been fully understood but it has been revealed that PARP1 interacts directly with ORF2, and the retrotransposase domain is responsible for this process. The absence of one of the PARP1 or PARP2 proteins leads to a decrease in the retrotransposition by about 50%; the absence of both proteins or the RPA complex reduces L1 retrotransposition by 80% [40]. The ORF2 complex, which is formed in the region of integration into the genome and promotes reverse transcription, includes various proteins involved in DNA stabilization and enzyme processability [42]. The first of these proteins is the proliferating cell nuclear antigen factor (PCNA). ORF2 interacts with PCNA and this interaction is critical for retrotransposition [43]. RUVBL1 and RUVBL2 repair proteins are also required for L1 spreading and their absence leads to a decrease in the retrotransposition [43]. In addition, the nonsense-mediated decay factor UPF1 and the MOV10 helicase were detected in the L1 RNP. Interestingly, UPF1 knockdown increases the amount of mRNA and L1 proteins but simultaneously reduces the effectiveness of the retrotransposition [43]. Inhibitory activity against L1 was shown for MOV10. However, in a recent study, it was suggested that MOV10 may facilitate the attachment of UPF1 to L1 RNP [42,44]. Insertion of the full-length L1 copy is a rare event; usually, a new copy of the L1 element is truncated from the 5′UTR [26,45,46,47]. Double-stranded DNA repair factors XRCC6 (Ku70/Ku80), Artemis (DCLRE1C), and LigIV (LIG4) are involved in the truncation of a new copy of the L1 element [46] (Figure 2). The exact mechanism of their action remains unclear. It is suggested that XRCC6 can facilitate the attachment of ORF2 to the overlying targeted DNA, thus accelerating the completion of the retrotransposition process and leading to truncation [46]. After integration of the reverse complement strand of the new L1 copy, the second strand of DNA breaks and the first strand of the new L1 copy is synthesized using the host cellular enzymes involved in both DNA replication and reparation [34,40]. Retrotransposition can occur using an alternative endonuclease-independent (EN-independent) mechanism in p53-defective cells or cells containing mutations in the non-homologous end-junction (NHEJ) genes during DNA repair, which apparently uses DNA breaks to initiate transcription [45,48,49,50].

## 4. L1 Evolution

L1s belonging to the LINE class of mobile genetic elements are found in the genomes of animals and plants [51,52]. Although animal L1 is found in the genomes of some protostomes, the history of gradual accumulation and the evolution of modern L1s can be traced at the level of deuterostomes, possessed by three highly divergent groups: a united species from echinoderms to teleost fishes; non-mammal vertebrates; vertebrates from fish to mammals [52,53].

### 4.1. LINE Evolution in Deuterostomes and Non-Mammals

A high diversity of ancient L1 families was found in the lancelet, sea urchin, and tunicates, and despite their variability, they make up a small proportion of the repeated sequences [53,54]. Mobile elements are in constant competition with each other and with factors limiting their activity in the cell, and they try to maintain the ability to spread in the genome and increase the number of copies by capturing new genomic loci. However, this battle is not always successful considering that in most bony fish genomes, although there are exceptions, the number of LINEs is not large compared to other mobile elements, yielding to DNA transposons [54,55,56]. On the contrary, in the known representatives of living jawless fishes, cartilaginous fish, coelacanths, and lungfishes, the number of LINEs is not inferior to other classes of DNA repeats and constitutes 25–50% of all repeats in the genome [54,57,58,59,60,61,62]. Interestingly, increased diversity of the L1 families is observed in fish [53]. Moreover, the highest diversity of L1 was found in African coelacanth. Nonetheless, the most successful LINEs in this group are still CR1 and L2 [56]. A high diversity of L1 was observed in amphibians, although, similar to that in bony fish, the number of LINEs remains small and most of them are either DNA transposons or LTR [53,54,58]. In reptiles, except green anole, several LINE families (CR1, BovB, L2) are evolutionarily successful, the activity of which continues to persist in the genomes, and their amount increases relative to other repeats [55,63,64,65,66,67,68]. The genome of tuatara is distinguished by a variety of repeats in which L2 is the most successful group [68]. The most widespread CR1, completely displacing L1, is in the genomes of turtles, crocodiles, and birds. The success of these elements was facilitated by the highly conserved hairpin structure and octameric microsatellite motif at their 3′UTR [65,69,70,71]. In the avian genomes, there was a sharp decrease in the genome size and number of repeated sequences. LINE/CR1 are the remaining bulk [71].

### 4.2. LINE Evolution in Mammals

In mammals, many LINEs lost the ability to spread due to various mutations and truncations of full-length copies [64,72]. Only one family of LINEs remains active. The most successful group of mobile elements in mammals is L1. An exception is the group of monotremes, which have no L1 sequences [52]. Thus, in platypus, L2 is the most prevalent of LINEs [73]. Metatheria (marsupials) and eutheria (placental mammals) have similarities in the composition and evolutionary tendencies of their mobile elements. In the genomes of most mammalian species, L1 becomes the most successful and active group, while many ancient repeats gradually disappear from the genome in process of evolution.; Intrestingly, that some enhancers and ultra-conserved elements are originated from ancient retrotransposon repeats [55,74,75]. Active L1s are species-specific genomic elements. Nevertheless, their structure is similar in all mammals and the greatest differences involve the non-coding region 5′UTR, the size of which varies greatly in different species [64]. 5′UTR changes play an important role in the interaction with cellular transcription factors that regulate L1 expression. Of the L1 encoded proteins, ORF1 differs in variability, while ORF2, on the contrary, is conservative [64]. Differences and evolutionary trends of L1 elements in mammals have been described in some animal groups. For example, some bats, similar to flying birds, have a decrease size of genomes and are characterized loss of active L1 elements [71,76]. The L1 extinction is also observed in certain mammalian species that are not adapted to flight. The disappearance of L1 activity was noted for *Spermophilus tridecemlineatus* from the superorder Afrotheria, perissodactyls, and sigmodontine rodents [77,78,79,80].

L1 is active in rats and mice. However, the accumulation and activity of mobile genetic elements of the widely studied mouse (*Mus musculus*) differ from the general tendencies of mammals, including humans and other primates, because LINE elements are quantitatively inferior to LTR repeats in its genome [81]. However, L1 makes up about 20% of the murine genome and L1Md is currently active. LINEs account for about 23% of the rat genome [82]. In addition to the traditional L1, the rat genome acquired the activity of HAL1 (HALF-L1) elements, the shorter version of L1 elements. In follow up the integration into the genome the HAL1 elements retain their internal promoter, that is othen truncated in case of integration of full-length L1 elements.

### 4.3. LINE Evolution in Primates

Primates separated from other ancestral mammals about 90–65 million years (myrs) ago and are characterized by the distribution of the L1PA-L1PB families [83,84,85]. Comparative evolutionary analysis of L1 revealed different trends in discrete primate species [86]. In most primate species, L1 is the most active family capable of self-propagation in the genome, as well as the most capable of contributing to the amplification of SINE elements, the copy number of which in genomes reaches the maximum of all dispersed repeats. In most species of New and Old World primates, the L1 remains active. Only in New World South American spider monkeys the absence of L1 activity was found [87,88]. The evolutionary history of the Old World primates began approximately 21–25 myrs and is associated with the distribution of L1PA6—L1PA5 elements [83,89,90,91]. L1PA5–6 elements, which are evolutionarily closest to their modern active L1 subfamilies, are most widely distributed in the genomes of monkeys (Cercopithecoidea) [89,90]. Interestingly, the greatest differences in the number of primate L1 were found among the Cercopithecoidea. For example, the baboon has the highest L1 amplification rates in the genome compared to other primates. On the contrary, the green macaque has the lowest number of L1 repeats compared with other primates [86].

The branch of great apes split off about 26 myrs ago [92]. Among the great apes, the largest number of L1 insertion loci was found in the orangutan. Moreover, the number of LINEs in orangutan genome significantly dominates over other families of dispersed DNA repeats, while in other primates, SINE insertions are most common [86]. Compared to other primates, the number of LINE insertions in humans is not large. However, the largest number of currently active LINE elements was found in the human genome [86]. Thus, the gorilla genome harbors twelve intact full-length gorilla-specific L1s belonging to the L1PA2 subfamily [93]. In chimpanzees, L1Pt-2 are active and only nine copies are full-length elements with intact ORFs [94]. In contrast, in humans, the active family is the L1HS, consisting of several subfamilies, namely pre-Ta, Ta-0, Ta-1, Ta1-d, and Ta1-nd [9,95] [, of which about 146 copies are active [5]. Moreover, comparative analysis showed that the activity of human L1 copies is significantly higher than that of chimpanzees [90,94].

### 4.4. LINE Evolution in Ancient and Modern Humans

A number of studies have shown that the accumulation of loci containing L1 repeat insertions is not random but occurs in accordance with functional significance. Thus, L1 insertions are more often retained in the trans-orientation relative to the gene, while insertions in the cis-orientation are washed out from the genome [96,97]. The evolutionary trends of L1 in the Homo branch are of great interest. However, the genomic architecture of L1 elements in ancient humans (*Homo sapiens sapiens*) and related subspecies, ancient hominids, (Neanderthals and Denisovans) are poorly understood due to the difficulties of genomic mapping of repeat elements using short reads available from the sequencing of ancient DNA. Nevertheless, several studies carried out an analysis of the mobile elements, which showed the presence of introgression of the L1 insertion loci of ancient people in the DNA of modern people, the nature of which corresponds to the same for SNV [98,99]. Moreover, in the genomes of ancient hominids, the sequences corresponding to the most active L1Ta1d mobile elements of the modern human genome were determined. Thus, the origin of L1Ta1d could have occurred in the common ancestor of ancient hominids and modern humans more than 800 thousand years ago [98]. An analysis of the insertion loci in genes in ancient people and modern humans showed that most of the repeat insertion loci specific to modern humans, including L1, originated in the genes that are highly expressed in the brain and are involved in neuronal maturation [99].

Analysis of L1 insertions in modern world human populations of the Phase3 data release project 1000 Genomes, which included 2.5 thousand individuals from 26 populations, reveals 2.91 thousand polymorphic L1 loci [100]. It was found that the majority (over 93%) of the identified loci of active retrotransposons (L1, Alu, and SVA) have a low population frequency of less than 5%. Moreover, such lowfrequency of insertion loci have substantial geographic differentiation. In support of this, in a recent study with a significantly smaller number of individuals (296 individuals) but greater population diversity (146 populations) from the Simons Genome Diversity Project (SGDP), a relatively large number of 1.886 thousand polymorphic unreferenced L1 loci were identified [101]. In both studies, the number of L1 polymorphic loci is 6–10 times lower than the Alu polymorphic loci but exceeds 3.5–4 times the number of SVA element polymorphic loci. The polymorphism of the insertion loci of active retrotransposons reflects the evolutionary aspects of modern populations and the migration processes of the world [100,101]. The greatest diversity is observed in Africans who are evolutionarily basal in world populations [100,101]. A decrease in heterozygosity is observed in populations of Eurasia and a minimum value was found in Native Americans [101].

### 4.5. LINE Evolution and Host Regulation

The L1 regulatory factors are evolved along with evolution of L1 elements. *APOBEC3* protein family and the Piwi-interacting RNA (piRNA)-signaling pathway are involved into the cellular defense mechanisms against the uncontrolled spread of L1 (see *Regulation of L1 Activity*). One of the most susceptible proteins to strong evolutionary selection, amplification, and divergence in mammalian genomes is the *APOBEC3* subfamily of antiviral factor genes [102,103]. High divergence of *APOBEC3* was noted in the genomes of bats and primates [104,105,106]. Interestingly, other closely related genes belonging to the *AID*/APOBEC family have lower evolutionary rates in mammals [107]. High evolutionary rates are also observed for the piRNA pathway, many genes of which are under the influence of positive selection [108]. The different regulatory pathways capable to repress L1 elements have been evolved reflecting the constant battle between mobile elelements and the cellular host defence [109,110,111]. The difference in expression of genes involved in host defence pathways of mobile elements between animal species plays an important role in the effectiveness of L1 inhibition. As shown in one study, there is a higher expression level of *APOBEC3B* (also known as *A3B*) and *PIWIL2* genes in human pluripotent stem cells, compared to the closest non-human primates (*Pan troglodytes* and *Pan paniscus*). The study showed that L1 silencing in human cells is more efficient as compared to chimpanzee cells [112].

Further, the factors regulating L1 elements are considered in detail.

## 5. Regulation of L1 Activity

**Figure 3:**
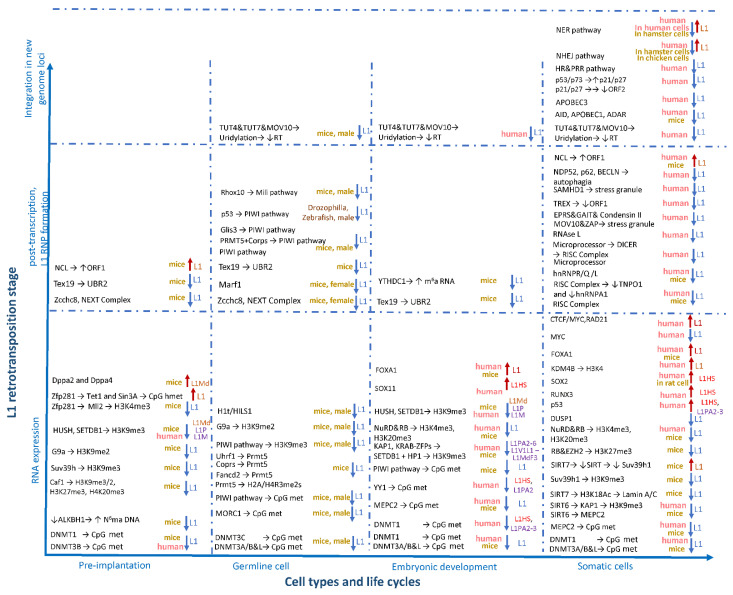
Factors affecting the activity of L1 retrotransposons during ontogenesis. The factors involved in L1 regulation are grouped horizontally in accordance with the stages of prenatal development (pre- and post-implantation period and in germline cells) and the postnatal period (somatic cells), as well as vertically depending on the stage of the retrotransposition process (expression, L1 RNP formation, and integration into the genome).

The process of the regulation of L1 activity throughout ontogenesis is complicated. In most cells, L1 activity is inhibited at all stages of the retransposition process in various ways: by decreasing the availability of DNA using DNA methylation [110,113,114], histone modifications, and heterochromatin formation [110,115,116]; through post-transcriptional inhibition by degradation of new RNA copies of L1 [117,118]; through repression of ORF1 and ORF2 translation; through the binding of L1 RNPs and the obstruction of their transportation to the nucleus [119,120,121,122]; and, at the last stage of integration for a new copy of the L1 element into the genome, through using DNA repair mechanisms [120,123,124,125,126] (Figure 3). In the process of organism ontogenesis, changes in the regulation of L1 activity occur. Thus, at the stage of formation of germ cells and mature germ cells, L1 poses a great threat to the future organism and, therefore, is thoroughly repressed by cells [127,128]. Most experimental knockouts of factors involved in the L1-silencing in germ cells lead to their death and infertility [129]. In the early stages of embryogenesis, activity of L1 is also dangerous for the developing organism and, therefore, is repressed [130,131,132]. Some changes occur in the pathways of L1 repression during embryogenesis and the activity of L1 elements increases at certain stages [133,134]. L1 elements are mainly repressed in somatic tissues of a mature organism, but increased L1 activity is noted for some pathologies including cancer as well as autoimmune and neuropsychiatric disorders [135]. With normal ageing, changes in the number of insertions of L1 are insignificant [136]. However, in some tissues, especially in the brain, L1 is not completely suppressed and L1 retrotranspositions can be activated [137,138,139].

### 5.1. Regulation of L1 in the Early Stages of Embryogenesis

The regulation of the L1 element activity in germ cells and during embryogenesis has been most thoroughly studied on model objects such as mice (Figure 3). DNA methylation is one of the main mechanisms of epigenetic regulation in the genome. Methylation during embryogenesis is provided by the methyltransferases *DNMT1* and *DNMT3B*, the knocking out of which leads to the death of mouse embryos [140,141] and human embryonic cultured cells [114]. DNA methylation undergoes changes during the process of embryonic development. The highest methylation level in mice is observed at the zygote formation stage, after which the first short-term decrease in methylation DNA level occurs, the minimum of which is reached at the 8-cell embryo stage [133]. It has recently been shown that global DNA hypomethylation during this period supplies the factors Dusp6 and Corp1 [142]. According to some data, at the stage of the preimplantation embryo, mice showed high levels of expression of various retrotransposons, particularly L1, the peak expression of which falls on the 2-cell embryo stage, after which the expression decreases [143,144,145]. However, an increase in the number of new L1 insertions was not detected during the experiment [145]. Recently, the paralogous transcription factors *DPPA2* and *DPPA4* have been shown to stimulate the expression of the active L1 families (L1Md_T and L1Md_A) and the *Dux* gene (MERVL repeat transcription factor) in mice at the 2-cell embryo stage [134]. The lack of *DPPA2* and *DPPA4* leads to the loss of the active chromatin marker H3K4me3 and gain de novo DNA methylation of developmental genes and young L1 [142]. At present, it is not known what function L1 performs at this early stage in the development of the organism. According to some reports, activation of L1 expression may affect chromatin availability and is necessary for the transition from the 2-cell embryo stage to the 4-cell embryo stage [145]. The interaction of L1 with nucleolin and Kap1/Trim28 leads to the inhibition of the transcription factor *Dux* and to increased expression of rRNA [146].

However, the L1 activation is short-term. In hypomethylated preimplantation mouse embryos, the inhibition of different families of retrotransposons occurs by histone methylation H3K9me3, H3K9me2, H3K27me3, and H4K20me3, mediated by histone chaperone chromatin assembly factor 1 (Caf-1) [115]. The knockout of this factor leads to the activation of various retrotransposons before the formation of the blastula and to the death of embryos at the morula stage [115]. In a recent study, the authors identified a total of 29 chromatin markers involved in the repression of retrotransposons in a murine embryonic stem cell culture [116]. For active L1 (L1Md), the highest enrichment with several histone markers was revealed including repressive (H3K9me3 and H3K9me2) and activating (H3K9ac9 and H3K56ac) modified histones [116]. To date, several methyltransferases have been identified that are involved in the histone methylation in the region of retrotransposons in murine embryonic stem cells. Arginine N-methyltransferase *Prmt5* plays an important role in the repression of retrotransposons in primordial germ cells and in preimplantation embryos in mice. Despite repression of L1 in germ cells, in mouse preimplantation embryos, *Prmt5* implements the control of IAP elements by histone methylation H2A/H4R3me2s, migrating from cytoplasm to the nucleus during the period of 2.5–3.5 embryonic days (E2.5–3.5) [130]. Methylation of H3K9 histones is carried out independently from each other using methyl transferases Suv39h, *G9a*, and SETDB1 [147,148,149,150]. *G9a* plays an important role in the mono and dimethylation of H3K9me1/me2 histones at an early stage of embryo development and germ cells, and its knockout leads to the death of mouse embryos between E9.5 and E12.5 [148]. Suv39h performs trimethylation of H3K9me3 histones directly in the regions of the L1 and ERV promoters [147]. There are two *Suv39h1* and *Suv39h2* genes in the mouse and human genomes that replace one another, and only double knockout of the genes leads to mortality after E12.5 [151,152]. Methyltransferase SETDB1 is mainly involved in the repression of IAP mice [149,150]. However, regulated by the factor TRIM28, SETDB1 also represses certain families of murine L1: L1V1L1–L1MdF3 families of origin age of approximately 5.57–3.77 myr [109]. Repression of active evolutionarily young copies of L1 (as well as copies inactive and older than 13 myr) in mice is carried out using the regulatory complex HUSH. The HUSH complex consists of three proteins, namely TASOR, MPP8, and Periphilin, that promote the formation of H3K9me3 histones methylation and the formation of heterochromatin [153,154]. The lack of the TASOR component of the HUSH complex leads to the death of mouse embryos before the completion of gastrulation [155]. Silencing of mobile elements can also occur due to a decrease in the number of markers of active chromatin. Thus, histone methylation of active marker H3K4me3 is provided by Mll2 methyltransferase, which is regulated by the transcription factor Zfp281 [156]. To repress active repetitive elements, Factor Zfp281 restricts Mll2 through regulating the activity of Tet1 and Sin3A hydroxymethylases in L1 promoter regions [156]. In addition to DNA methylation and histone methylation, a decrease in L1 expression and the formation of heterochromatin in mammals is facilitated by another evolutionarily ancient method of DNA modification: N6-methyladenilation [157,158]. A study in mice revealed that this marker suppresses L1 in mouse embryonic stem cells [159]. Marker N6-methyladenine is located mainly in areas of young L1 (<1.5 Ma). The presence of this marker leads to their inactivation. A deficiency of the N6-methyladenine demethylase enzyme, ALKBH1, leads to transcriptional inhibition [159]. At the post-transcriptional level, RNA transcripts can also be labeled with N6-methyladenosine, leading them to be silenced [160]. This manner of transcript inhibition can be used by mouse embryonic cells to repress LINE1 and other retrotransposons (IAP, ERVK, etc.) [161,162]. A lack of YTHDC1, a factor that initiates silencing by recognizing N6-methyladenosine modification and transcript binding, leads to the transition to the 2-cell embryo stage [161,162]. Post-transcriptional degradation of L1 in early embryos can occur due to the nuclear exosome targeting (NEXT) complex, the central factor of which is *Zcchc8* [131]. Knockout of the *Zcchc8* factor in mice increases chromatin availability and L1 expression, resulting in impaired blastula formation and decreased survival of homozygous knockout mice [131].

### 5.2. Regulation of L1 in Germ Cells in Mice

**Figure 4:**
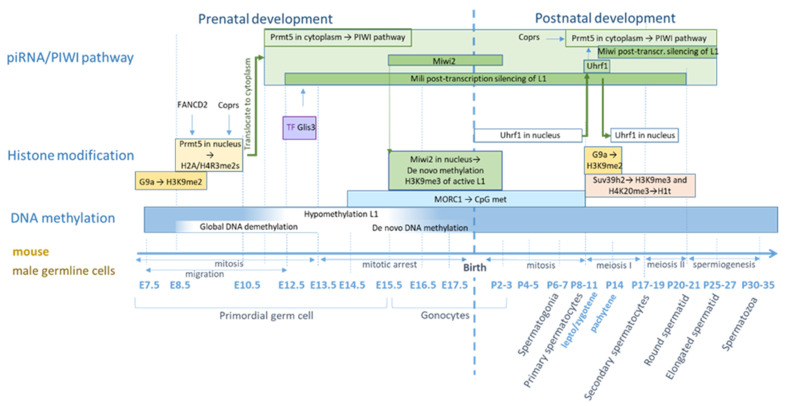
Scheme of L1 repression during the formation of male germ cells in mice. The factors involved in L1 restriction in male germ cells are depicted according to their activity at certain stages of pre and postnatal mouse development. The *X*-axis shows the age of the mouse and the corresponding types of male germ cells.

The formation of primordial germ cells (PGS) in mice occurs on E7.25 (Figure 3 and Figure 4) [163,164,165,166,167]. In PGS, DNA undergoes global demethylation, which occurs on day E8.5 of embryogenesis and lasts until E13.5, when PGS are grouped into the genital ridge [168,169,170,171]. Demethylation leads to activation of IAP repeats, however, L1 remains silent at these stages [170]. After E13.5, the methylation profile of male and female germ cells is different [171]. In mice male germ cells, DNA methylation occurs due to Dnmt3A and 3B DNA methyltransferases, as well as due to the Dnmt3L protein, which does not have enzymatic activity but is an important auxiliary factor [172,173]. De novo methylation of evolutionarily young L1 and IAP families occurs using Dnmt3C, which is a duplicated copy of Dnmt3B in the rodent genome [174,175]. Methylation is initiated shortly after E13.5 and peaks after the birth [176]. The remethylation of L1 in male germ cells occurs briefly from E12.5 to E15.5 [132,177,178].

One of the most studied mechanisms of L1 inhibition in male embryonic germ cells is the mechanism that involves the Piwi-interacting RNA (piRNA)-signaling pathway, acting at the transcriptional and post-transcriptional levels [179,180,181]. piRNAs are small non-coding RNAs, 24–32 bp long, that specifically bind to the Argonaute proteins of the PIWI subfamily [182,183]. The PIWI family of proteins interacting with piwiRNA includes four proteins, namely PIWIL1-PIWIL4. Only three proteins are present in mice: *Miwi* (PIWIL1), *Mili* (PIWIL2), and *Miwi2* (PIWIL4). In contrast, in humans, there are all four proteins: HIWI (PIWIL1), HILI (PIWIL2), HIWI2 (PIWIL4), and HIWI3 (PIWIL3) [184]. L1 degradation in hypomethylated embryonic germ cells can occur through direct interaction with the piRNA–PIWI complex with the new L1 RNA copy and its subsequent degradation. The piRNA–PIWI complex can also mediate DNA methylation. This complex is *Miwi2*–piRNA in the mice embryonic male germ cells [180,185,186]. Moreover, the piRNA/PIWI-signaling pathway is necessary to maintain a high level of H3K9me3 histone repression modifications in L1 regions [179]. Damages in many genes involved in the piRNA/PIWI-signaling pathway (including *Mili* and *Miwi2*) are associated with L1 activation of retrotransposons, impaired cell formation, and infertility. As previously mentioned, *Prmt5* is involved in the inhibition of retrotransposons, including L1, in PGS [130,187]. The control of the epigenetic regulation of *Prmt5* occurs using two pathways: (1) the methylation of histones H2A/H4R3me2s in the nucleus and (2) the enzyme entrance into the cytoplasm and activation of the piRNA/PIWI-signaling pathway [130]. In PGS, during the E8.5–10.5 period, Prt5 methyltransferase is located in the nucleus, participating in the methylation of histones H2A/H4R3me2s in the regions of IAP and L1 elements [130]. Then, at approximately E11.5, the *Prmt5* enzyme migrates into the cytoplasm and provides arginine methylation in Piwi proteins, which is necessary for binding to proteins containing the Tudor domain [187]. *Prmt5* knockout in PGS results in complete sterility of the female and male individuals [130]. Recently, *Prmt5*-dependent histone methylation has been shown to be catalyzed by the *Fancd2* factor, one of the most important factors for homologous DNA recombination and repair involved in the suppression of L1 in somatic tissues [188]. Knockout of the *Fancd2* factor leads to a decrease in *Fancd2*-catalyzed H2A/H4R3me2s markers in the L1 region at E10.5 [188]. Additionally, Corps factor (Copr5) is required to activate *Prmt5* [189,190]. In mice PGS, *Mili* protein expression begins from E12.5 and lasts the longest until around the spermatid stage in adult testicles [190,191]. *Mili* is located mainly in the cytoplasm and is required for the primary processing of piRNA, secondary piRNA biogenesis, the ping-pong cycle, de novo DNA methylation, and post-transcriptional inhibition of L1 retrotransposons [180,192]. Along with *Mili*, Mov10L1 [193], *Tdrd1* [194], and *Tdrkh* (Tdrd2) [195] are also involved in the primary processing of piRNA, for which it is well shown that their defects lead to L1 activation, arrest of meiosis, and sterility. A recent study has shown that the factor of positive regulation of *Mili* is the *Rhox10* transcription factor [196]. Although the *Rhox10* gene is present only in the genomes of mice, the contribution of the Rho family to the regulation of Piwil2 is evolutionarily conservative according to the authors of the publication [196]. In the interval between E12.5 and 13.5 in mice, during hypomethylation, the testis-specific transcription factor Glis3 is activated. This factor provides the inhibition of various retrotransposons, especially L1 and IAP [197]. During late embryonic development and in the first few days after birth, E15.5-P3, expression of *Miwi2* is observed, the protein complex of which induces de novo DNA methylation that inhibits the spread of L1 [180,185]. In addition, *Miwi2* provides trimethylation of the H3K9me3 histones of active full-length L1 and LTR elements [179]. Lesions in the genes *Tdrd1* [194], *Mvh* [198], *Tdrd12* [199], *Fkbp6* [200], and *Gtsf1* [201] lead to disruption of the complex formation and dislocation of *Miwi2* from the nucleus to the cytoplasm, and also lead to the impairment of various retrotransposons and L1-silencing in the nucleus. The Exd1 factor interacts with *Tdrd12* and, with its partial deficiency, also leads to both the disruption of *Miwi2* biogenesis and activation of L1 [202,203]. Recently, it was shown that in the perinatal period (E16.5–18.5), the expression of factor *Tex15* occurs, which affects the methylation of only active L1 and IAP retrotransposons. The exact role of factor *Tex15* in such a short period of time is not completely clear. An absence of it does not disturb the biogenesis of piRNA but leads to a decrease in the expression of the *Gtsf1* factor associated with both the *Mili* and *Miwi2* proteins [201,204]. Regardless of the piRNA/PIWI-signaling pathway, the Morc1 factor regulates the methylation of retrotransposons from the late embryonic stages (after E14.5) to the first meiosis in male embryonic germ cells; a deficiency of this factor leads to demethylation of the L1 transcription initiation regions and to an increase in its expression at the E16.5 stage [205]. Morc1 deficiency also leads to the expression of other retrotransposons (LTR and IAP) [205].

In mature mice, L1 is inactive in the stem cells of the testes–spermatogonia but is active in spermatocytes [193]. L1 suppression in spermatogonia of mice is carried out using several mechanisms due to DNA methylation and the piRNA/Piwi-signaling pathway, as well as due to histone methylation. DNA methylation of the L1 promoter regions during the period of postnatal development and up to the stage of meiosis of primary spermatocytes is performed by the Morc1 factor. The onset of meiosis I in male germ cells is associated with epigenetic changes in the profile of DNA and histone methylation. In the first stages of meiosis during the period of leptotene and zygotene, methylation of histones H3K9me1/2, carried out by methyltransferase *G9a*, plays an important role in L1 repression [206]. In addition, *Mili*, which has the longest expression period in the pre and postnatal period, makes a large contribution to the repression of retrotransposons [192]. During this development period, the deficiency of one of the pathways is compensated by the other. An increase in L1 activity is observed only with the double knockout of *G9a* and *Mili* [206]. *Mili* is known to repress retrotransposons at the post-transcriptional level. *Mili* can also participate in DNA methylation in combination with *Miwi2* in the prenatal period [180,207]. A recent study showed that *Mili* can interact with *Tex15* and this is necessary for the methylation of retrotransposons in germ cells [204]. Additionally, from the beginning of the leptotene stage to the stage of round spermatids, the expression of *Suv39h2* methyltransferase is observed, which is involved in the methylation of histone H3K9me3 [151,208]. Thus, a recent study showed the association of the histone of the H1t linker with repressive histone markers H3K9me3 and H4K20me3 in the region of L1 and LTR repeats, and suggested the participation of the piRNA/PIWI-signaling pathway in the formation of heterochromatin [209]. In an earlier study on rats, the presence of a spermatid-specific histone linker (HILS1) was also found, which promotes the formation of higher-order chromatin structures in L1 regions as well as the enrichment with histone markers H3K9me3, H4K20me3, H4K5ac, and H4K12ac [210]. At the beginning of meiosis I, at the stage of prophase I, a change in the localization of the Uhrf1 factor is observed. The Uhrf1 moves from the nucleus to the cytoplasm, where the factor arrives in the period from leptotene to early pachytene, and then migrates back to the nucleus [211]. The Uhrf1 factor is involved in the regulation of the epigenetic profile of both DNA methylation via *DNMT1* and the formation of histone repression markers H3K9me3 during mitosis interacting with various factors that modulate histones [211,212,213,214]. Recently, it was shown that at the prophase stage of meiosis I, Uhrf1 is necessary for the interaction with *Prmt5* and activation of PIWI proteins at the pachytene stage, and its absence leads to the activation of L1 and AIP retrotransposons, resulting in a decrease in DNA and histone methylation, as well as a deficiency in the piRNA/PIWI-signaling pathway, leading to defective meiosis and sterility [130,211]. It has also recently been shown that in adult testicles, Corps is required for *Prmt5* activation and its knockout leads to a decrease in *Miwi* and L1 activation, disrupting the maturation of spermatogonia in spermatids and leading to infertility [190]. At the pachytene stage, many factors participating in the piRNA/PIWI-signaling pathway begin to be expressed, including the *Miwi* protein, which is characteristic of mature testicles [207,215]. The lack of piRNA/PIWI-signaling pathway factors is reflected in the efficiency of L1 repression in testicles. Thus, the deficiency of the *Pnldc1* and *Tdrkh* genes involved in the primary processing of piRNA leads to L1 depression and spermatogenesis defects [216,217,218]. It is worth noting that at the last stages of sperm maturation in mice, the L1 DNA methylation undergoes changes. A recent study showed that, starting from the round spermatid stage, the regions of active L1 promoters are enriched with 5-carboxymethylcitazines (5-caC), which are considered as a marker of active demethylation [219,220].

Control of the retrotransposons activity in female germ cells is different from male germ cells. In the precursors of female germ cells, L1 demethylation occurs gradually starting from E10.5 to E15.5–17.5 [132,177]. Despite the expression of piRNA/PIWI-signaling pathway proteins in female germ cells, its deficiency does not affect the development of oocytes [221,222,223]. There is also a hypothesis that the absence of the piRNA/PIWI pathway can be compensated by the RNA interference pathway [224,225]. However, a recent study showed that the absence of both signaling pathways, although causing an increase in the number of L1, does not interfere with the development of female germ cells in mice [226]. Studies published in the last two years have shown that control of the L1 activity of female germ cells (oocytes) in mice is carried out at a post-transcriptional level that promotes the degradation of L1 transcripts (Figure 3). The first suppressor L1 was found as a factor of meiosis arrest female 1 (Marf1) in mice female germ cells [227]. Marf1 has ribonuclease activity and provides the degradation of single-stranded RNA, preventing the spread of retrotransposons [118]. It has also recently been shown that in oocytes, in mice, in the early embryonic stages, L1 inhibition occurs due to post-transcriptional degradation of RNA using the nuclear exosome targeting (NEXT) complex and the *Zcchc8* factor [131]. The survival rate of maternal *Zcchc8* knockout mouse embryos is reduced, the expression of L1 and MERVL is markedly increased at the 4-cell embryo stage, and only half of the embryos reach the blastocyst stage and continue to develop [131].

### 5.3. Other Factors Controlling L1 in the Embryonic Development of Mice

The list of factors in the regulation of retroelements in the process of embryonic development is constantly updated. Factor *Tex19.1* and its paralogue *Tex19.2* with a high level of expression in embryonic pluripotent cells, the placenta, and testicles are specific for mice and rats, and are not found in humans. Controversial data were obtained regarding factor *Tex19.1*. According to some reports, *Tex19.1* affects the regulation of MMERVK10C repeats and does not affect L1 expression in male germ cells and placenta [228,229,230]. In contrast, in other studies, it was shown that *Tex19.1* can interact with L1-ORF1 and stimulate both polyubiquitinylation and proteasome-dependent degradation of L1 using UBE2A/UBR2 in the placenta and embryonic stem cells [229,231].

### 5.4. Features of L1 Regulation during Human Embryonic Development

In a recent study, L1HS activation was shown in preimplantation embryos and placenta in humans with an expression peak on day E8 of the embryonic development [232]. Previous study showed there was no short-term decrease in methylation and L1HS activation in the early stages, but there was a decrease in methylation and an increase in L1 expression in the placenta in the third trimester compared to the first trimester, probably associated with a decrease in *DNMT3B* expression [233]. Features of L1 activity in various tissues during human embryonic development remain poorly understood (Figure 3). The largest number of somatic mutations were detected in the human brain compared to other tissues such as the heart and liver [113,139,234,235,236]. DNA methylation in humans, as well as in mammals, is carried out by three main methyltransferases: *DNMT1*, DNMT3A, and *DNMT3B* [237,238]. DNMT3A and *DNMT3B* are mainly responsible for de novo methylation in the region of pericentromeric heterochromatin [141], while *DNMT1* maintains an inherited DNA methylation profile in dividing cells [239]. Unlike the DNMT3A and *DNMT3B* enzymes, whose knockout does not impair the viability of human embryonic culture cells, *DNMT1* knockout in a culture of human embryonic cells leads to their immediate death [114]. Despite this, in human neural progenitor cells (hNPCs), *DNMT1* knockout did not lead to their death but rather increased the expression of young L1HS and the closest L1 families (L1PA2 and L1PA3). In contrast older L1 families and other repeats of HERV, SINE, and SVA, despite the absence of methylation, remained silent [240]. In neuronal progenitor cells of mice and humans, the transcription factor involved in the DNA methylation of the L1 promoter region is MePC2 (methyl-CpG-binding protein 2) [241]. MePC2 selectively represses L1 elements specifically but not other retrotransposons [242]. On the culture of neuronal progenitor cells of mice and humans, it was shown that mutations in this gene lead to an increase in L1 activity [113]. The L1 5′UTR region contains several binding sites for transcription factors, one of which is zinc finger protein Yin Yang 1 (YY1) [243]. A high expression level of the transcription factor *YY1* is observed in the brain during embryonic development [244]. The YY1 binding site is the most evolutionarily conserved in many L1s and has a dual role in regulation as an activator and repressor [237,238,239]. According to one of the earlier studies, YY1 has only minor effects on promoter activity but is required for proper initiation of L1 transcription [27]. At the same time, a recent study showed that in human embryonic stem cells (hESCs), neural progenitor cells, hippocampal neurons, brain and liver tissues, and younger L1 families (L1HS and L1PA2) are repressed using the YY1 transcription factor, which facilitates methylation of the L1 promoter [110]. A slight truncation of the L1 5′UTR region containing the YY1 site avoids repression [110]. The repression of older L1 families that rose to 26.8–7.6 Ma ago (L1PA3-L1PA6) in human embryonic cells is carried out through histone modification, which is regulated by factor KAP1 (TRIM28) [109,110]. KAP1 (KRAB-associated protein 1, tripartite motif protein 28 (TRIM28)) are cofactors of KRAB-ZFPs (DNA-binding Krüppel-associated box domain-containing zinc finger proteins) [149,245,246], the activation of which leads to the histone H3 lysine 9 trimethylation (H3K9me3) using methyl transferase SETDB1 (ESET) and the formation of heterochromatin using the heterochromatin protein 1 (HP1) [247,248,249]. Recently, it was shown that the HUSH complex is also involved in the regulation of SETDB1 [111,250]. The HUSH complex also recruits MORC2 ATPase to form heterochromatin [111,251]. The activity of the HUSH complex in humans contributes to the repression of old families of L1P and L1M retrotransposons in the introns of active genes [111]. DNA methylation and histone methylation are interrelated, thus, recently it was found that *DNMT1* recognizes heterochromatin markers H3K9me3 and H4K20me3, which profoundly impacts DNA methylation [252,253]. A low level of expression in the mammalian embryonic and adult brains is observed for PIWI family proteins. Whether the piRNA/PIWI-signaling pathway plays any role in the repression of human retrotransposons in brain is unclear [184,244,254,255,256]. However, in an experiment in mice, it was found that although the expression level of the a murine PIWI protein (*Mili*) is very weak, its knockout leads to hypomethylation of the regions of L1 promoters in the hippocampus and prefrontal cortex, and affects locomotor and cognitive function, causing hyperactivity and reducing anxiety [255].

### 5.5. L1 Regulation in Somatic Cells

In most somatic cells in the organism, L1 is repressed and its activation is associated with various pathologies, such as cancer and some autoimmune and neuropsychiatric diseases [257,258,259,260,261]. An exception is brain tissue, where L1 activity seems to be a common in neurons and glial cells [262,263]. The mechanisms of L1 regulation in somatic cells are mostly studied in cultured cells derived from human and mouse cancerous, stem, and embryonic tissues. Some molecular mechanisms such as DNA methylation and decreased chromatin availability for controlling the distribution of L1 are formed during embryonic development and persist during life [264,265,266,267]. For many factors it is currently not known whether they participate in the further repression of L1 in the process of postnatal development due to their expression in the tissues of the adult organism or whether they are limited to the stages of embryonic development.

**Figure 5:**
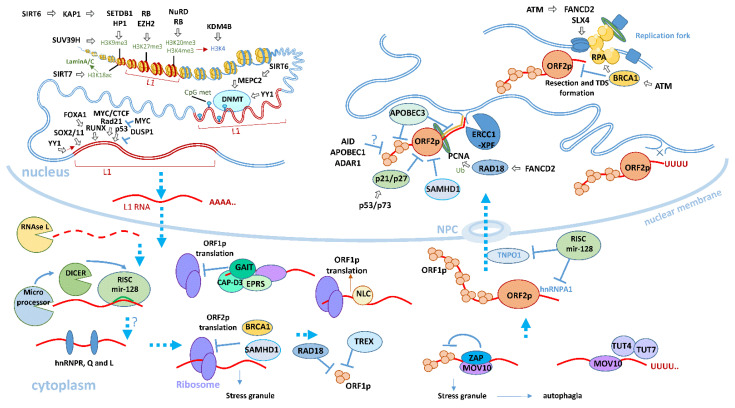
Scheme of L1 regulation at various stages of the process of retrotransposition in somatic cells. The upper left part of the figure shows the regulation of L1 expression: inhibition by decreasing chromatin availability due to DNA and histone methylation, and increased expression in the region of open chromatin and with the participation of transcription factors. In the cytoplasm, L1 is inhibited by microRNA pathways and interferon-activated factors involved in both the cleavage of the L1 transcript and in preventing the formation of L1 RNP (shown at the bottom of the figure). The binding of factors to the L1 internal ribosome entry site can both inhibit and promote the translation of proteins. Integration into the genome is impeded by both antiviral factors and factors of DNA repair and the cell cycle (shown in the upper right part of the figure). DNA repair factors are also required for the integration of a new copy into the genome.

Next, methods for the regulation of L1 elements specific to various somatic tissues will be considered (Figure 3 and Figure 5).

#### 5.5.1. L1 Regulation by Decreasing Chromatin Availability in Somatic Cells

In most normal somatic tissues, L1 is repressed by DNA methylation and specific histone modifications (Figure 5). Currently, several factors are known that regulate the availability of chromatin in L1 regions in somatic tissues, including *MEPC2*, KAP2, sirtuins, and the NuRD complex [109,110,113,267,268,269]. Changes in the L1 methylation profile are often found in various forms of cancer [257,258,270,271]. Alcohol, smoking, and narcotic substances can cause a change in the methylation profile and L1 activation [265,272,273,274,275,276,277]. The methylation profile can be changed in somatic tissues under influence of various exogenous and endogenous factors, including age-related changes or some diseases [278,279,280,281]. There is an opinion that prenatal immune activation may affect the methylation profile in offspring in the adult brain [282,283,284,285]. Recently, data were obtained suggesting that, in adult mice, prenatal immune activation results in a change in the methylation profile of L1 and *MEPC2* in the brain in the prefrontal cortex and striatum [264].

Sirtuin genes are associated with life span and longevity, and are involved in many cellular processes, such as DNA repair, telomere length regulation, anti-inflammatory processes, protection against malignanttumors, cell proliferation, ribosomal and protein synthesis, metabolism, and many other processes [286,287,288,289,290]. One of the functions of the *SIRT6* gene is the suppression of L1 elements, carried out in two ways [267]. In the first case, mono-ADP (mono-ADP) ribosylation of factor KAP1 occurs, which in turn stimulates the formation of heterochromatin. Another less studied pathway is possible through interaction with the transcription factor *MEPC2*, which is involved in the methylation of the L1 promoter region. Knockout of the *SIRT6* gene in mice causes a several-fold increase in L1 expression and significantly increases the number of retrotranspositions [267]. Recently, another sirtuin gene, *SIRT7*, has been identified, which also inhibits the activity of L1, mainly concerning young families capable of retrotransposition in both mouse and human cells [268]. Interestingly, the L1 suppression mechanism identified by the authors of the article is fundamentally different. *SIRT7* promotes the deacetylation of H3K18 histones and the formation of heterochromatin, which results in the association of L1 elements with the nuclear lamin A/C and their repression [268]. Repression of L1 using *SIRT6* and *SIRT7* can occur in various somatic tissues of the brain, liver, heart, and many others, including those involved in embryonic development [244,267]. A decrease in *SIRT6* expression is shown in mice with age, especially in the brain, which leads to an increase in L1 expression and the development of the inflammatory process [267,291,292]. *SIRT7* can also negatively affect the activity of *Suv39h1* methyltransferase through inhibition of SIRT1 [293], for which a decrease in histone H3K9 methylation in the L1 region was noted with age in muscles in mice [294].

In addition to sirtuins, the nucleosomal and remodeling deacetylase multiprotein complex (NuRD) can lead to the formation of heterochromatin in both somatic and embryonic tissues [269]. This complex includes dermatomyositis-specific autoantigen Mi2 (Mi2-β), retinoblastoma-binding protein 46 and 48 (RbAp46 and RbAp48), methyl-CpG-binding domain protein 2 and 3 (MBD2 and MBD3), and metastasis-associated 1 family member 2 and 3 (MTA2 and MTA3), whose knockout is critical for L1 repression [269,295,296,297]. The NuRD complex binds to the 5′UTR promoter of the L1 elements due to the DNA and ATPase-binding domains of the Mi2-β subunit [298]. Moreover, retinoblastoma (Rb) proteins play an important role in the stabilization of the NuRD complex, the knockout of which leads to the delocalization of Mi2-β in the cytoplasm, disrupting the complex structure and leading to L1 activation [269,299]. Retinoblastoma (Rb) proteins can also form a complex with EZH2, which promotes the formation of H3K27me3 heterochromatin markers in the regions of various repeats: simple, satellite, transposons, and retrotransposons, including L1 [300].

#### 5.5.2. Post-Transcriptional L1 Regulation in Somatic Cells

There is a large number of cellular factors involved in the restriction and degradation of L1 transcripts at the post-transcriptional level (Figure 3 and Figure 5). Some of these factors act specifically with respect to L1, for example, by RNA interference mechnisms, while the effect of others is related to interferon-induced protection in response to various foreign nucleic acids and dispersed repeats.

#### 5.5.3. RNA Interference and miRNA Factors

RNA interference is an effective way of specific inhibition of L1 elements in somatic cells. Inhibition of L1 in somatic cells is carried out using miR-128, which together with the Ago protein form the RISC complex [301,302,303].

L1 repression by miR-128 can be processed through different mechanisms: by direct binding to the L1 mRNA transcript in the ORF2 region, leading to subsequent degradation of the transcript [301]; and by inhibiting cellular factors that facilitate the L1 RNP transportation from the cytoplasm to the nucleus, namely transportin-1 (TNPO1) [302] and hnRNPA1 [303]. TNPO1 is a cellular factor involved in the transport of proteins from the cytoplasm to the nucleus [304,305]. The cellular factor hnRNPA1 stabilizes the polyA end of various transcripts, including L1 RNA, and is involved in the intracellular transportation as well as accompanies mature transcripts through the nuclear pore complex (NPC) [303,306]. Other heterogeneous nuclear ribonucleoproteins (hnRNPs), namely R, Q, and L, also participate in the L1 inhibition by another mechanism, the details of which remain unexplored. However, it is known that their effect occurs through binding to the internal ribosomal entry sites (IRESs) in the region of 5′UTR [307,308]. Several protein complexes (Microprocessor and Dicer) are involved in the process of miRNA maturation. A microprocessor consists of a DROSHA, an RNAse III enzyme and two DGCR8 molecules, and a double-stranded RNA binding protein [309,310,311]. It binds to the pri-miRNA hairpin structure and cleaves it, which then undergoes further cleavage with DICER, and, together with Ago proteins, forms the RISC complex [312]. There is an alternative mechanism in which the Microprocessor is also able to directly inhibit L1 by recognizing the pri-miRNA-like harpin structure in the region of the 5′UTR L1 transcript and cleaving it, which prevents further maturation of the L1 copy [313].

#### 5.5.4. Antiviral Factors

Intracellular antiviral defense mechanisms of the cells also extend to dispersed repeats (Figure 3 and Figure 5). In response to INF-induced stimuli, activation of intracellular factors of the innate immune response occurs. These include factors involved in the inhibition of viral nucleic acids, which are also involved in the inhibition of various retroelements, including L1 [119]. Although antiviral intracellular factors are activated in response to both viral nucleic acids and retroelements, the same factor can use different inhibition mechanisms against viruses and retrotransposons [314,315,316]. The effectiveness of the inhibition of L1 and the viral agents is also not the same [317]. Moreover, recent studies have shown that L1 can be activated in response to a viral infection and can stimulate the innate immune response [318,319,320]. L1 activity can also be suppressed by viruses. For example, it has been shown that HIV-1 suppresses L1 retrotransposition by binding to ORF2 using the Vpr protein included in HIV-1 [321] or the hepatitis C virus, which provokes the formation of ORF1 stress granules [322]. Defects in many L1 inhibitory antiviral immune response genes are associated with autoimmune diseases such as Aicardi–Goutières syndrome [323].

The following are various antiviral cell factors that inhibit L1 in somatic cells.

##### Interferon-Induced Factors Inhibiting the Formation of L1 RNP

In response to a viral infection, the interferon-induced ribonuclease L (2′,5′-oligoadenylate (2–5A) synthetase (OAS) -RNase L, RNAse L) is activated, which cleaves cellular and viral RNA [117]. The enzymatic activity of RNAse L has also been shown with regard to L1 mRNA and other IAP retrotransposons, which leads to a significant decrease in the ORF1 and ORF2 L1 proteins [324]. The cleavage of RNA molecules into small pieces using RNAse L leads to the activation of RIG-I (DExD/H-box helicase 58, DDX58) and MDA5 (interferon induced with helicase C domain 1, IFIH1) sensors, which in turn activate MAVS and stimulates both the production of interferon and the immune response [325,326,327]. Recent studies have shown that expression of L1 mRNA triggers an immune response through RIG-I and MDA5 sensors, and increases the level of interferon β INFB [318].

Many studies have confirmed that the strong L1 inhibitor is the MOV10 helicase RNA [215,328,329,330,331]. The mechanism of the MOV10 action remains to be fully understood but it is known that MOV10 forms a complex with other antiviral proteins, which, when interacting with L1 transcripts, forms stress granules that undergo further degradation [215,330]. Mass spectrometry of stress granules revealed other key proteins complexed with MOV10 helicase. One of these proteins is zinc finger CCCH-type containing antiviral protein 1 (ZAP or ZC3HAV1) [120,332]. ZAP forms a complex with MOV10 that binds to L1 RNA and blocks ORF1, which then leads to the formation of stress granules with subsequent degradation. Additionally, in a recent article, the interaction of MOV10 with ribonuclease H2 (RNASEH2) has been shown. This interaction can prevent the formation of RNA-DNA L1 heteroduplexes, which are necessary for retrotransposition [329]. Interestingly, another study showed the important role of RNase H2 in L1 retrotransposition. It is assumed that this enzyme degrades L1 RNA after reverse transcription and thus contributes to the completion of L1 integration in the genome [333]. Studies have shown that in various human cells and testes of mice, a new L1 RNA copy undergoes 3′UTR urinylidylation as a result of the activity of TUT4 and TUT7 uridine transferases; undergoes complexation with the MOV10 RNA helicase; and subsequently degrades [334]. The MOV10-ZAP complex inhibiting L1 contains another component of glutamyl-prolyl tRNA synthetase (glutamyl-prolyl tRNA synthetase, EPRS) [43,120]. The EPRS synthetase is able to bind RNA and is a component of various complexes, one of which is the γ-interferon-activated inhibitor of the translation complex (GAIT) [335]. The GAIT complex binds to mRNA and inhibits its translation by repressing the translation initiation factor eIF4G by attaching the L13a subunit of this complex. The interaction of eIF4G and L13a blocks the 43S preinitiation complex and inhibits translation initiation [336]. It was revealed that EPRS also interacts with the CAP-D3 subunit of the Condensin II complex. It is assumed that such interaction is necessary for the formation of the active GAIT complex with the L1 transcript and for the further inhibition of L1 ORF1 translation [337]. Despite the fact that activation of the GAIT complex occurs in response to interferon γ [338], inhibition of L1 by the Condensin II–GAIT complex occurs independently of interferon γ [337].

Three-prime repair exonuclease I (TREX1) is an antiviral enzyme activated in response to interferon type 1 and cleaves nucleic acids [339]. It is also involved in the inhibition of L1 and other retroelements but, similar to the MOV10-ZAP complex, it uses an exonuclease-independent mechanism. There are no exact data on whether TREX1 is part of the MOV10-ZAP complex, but in the same way as the enzymes described above, the L1 inhibition mechanism consists of binding the L1 ORF1 protein and changing its intracellular localization as well as its further removal [340]. TREX1 is located in the endoplasmic reticulum (ER) [341,342], where ER-dependent protein degradation occurs [343].

The SAM domain and HD domain-containing protein 1 (SAMHD1) are some of the most well-known protective factors of innate immunity, inhibiting the replication of viruses and participating in the repression of both L1 and other retroelements [344]. The mechanism of inhibition of L1 differs from the antiviral inhibition and is observed in dividing cells [316]. Unlike the MOV-ZAP and Condensin II–GAIT complexes that block ORF1, SAMHD1 interacts with ORF2; the details of this repression are not completely clear and various authors have presented several ways of inhibiting L1 [121,316,345]. In a cell, SAMHD1 is located mainly in the nucleus [346], where SAMHD1 binds to ORF2, inhibits the reverse transcription of L1, and reduces the expression of ORF2 proteins (ORF2p) [316]. In another study, despite the nuclear arrangement of the enzyme, a SAMHD1 mechanism of L1 inhibition in the cytoplasm was revealed, which, through phosphorylation of the translation initiation factor eIF2α and through a decrease in the interaction of translation initiation factor eIF4A/eIF4G, leads to the activation of stress marker factors G3BP1 and TIA1, as well as to the formation of large stress granule of L1 [347]. In another study, it was found that SAMHD1 binds to ORF2p at the stage of the L1 RNP functional complex; moreover, the inhibitory activity of SAMHD1 against L1 is regulated by phosphorylation of threonine 592, the dephosphorylation of which correlates with the repression of L1 [121]. In addition to this site, another polymorphic site S33 phosphorylation was identified, which contributes to the inhibition of L1 [348]. As shown in a recent work, SAMHD1 is a nucleocytoplasmic shuttling protein and thus is possibly involved in the inhibition of L1 in the cytoplasm and nucleus, and the presence of SAMHD1 in the cytoplasm contributes to a decrease in ORF2p [349]. RNA granules, in which L1 RNA transcripts are localized, undergo autophagy, facilitated by the NDP52 (CALCOCO2) and p62 (SQSTM1) receptors [122]. Knockout of these receptors, as well as autophagy factors of BECN1, leads to an increase in L1 [122]. One of the new studied alternative ways of inhibiting L1 is the activation of TRIM25alpha, a known antiviral factor [350]. It is assumed that the TRIM25alfa mechanism of L1 inhibition is similar to HIV-1 inhibition; thus, TRIM25a using its CC region, B-box, and SPRY domains forms multimeric lattices that promote the binding to L1 RNP in the cytoplasm, blocking further stages of L1 retrotransposition [351,352]. In addition, TRIM25a acts as a cytoplasmic L1 receptor and initiates an innate immune response through the activation of the AP-1 and NF-kB factors, leading to the downregulation of the L1 promoter [352].

##### Deaminases

The *APOBEC3* protein family is one of the first discovered cellular defense molecules against the uncontrolled spread of L1 [119,125,353,354]. In various human cell cultures, data were obtained showing that most of the proteins of the *APOBEC3* family (hA3A, hA3B, hA3C, hA3F, and hA3DE) have inhibitory activity against L1 [355,356,357,358,359]. Weak inhibitory activity or its absence was observed for hA3D [357], and conflicting results were obtained for two other hA3G and hA3H enzymes [356,357,358]. Inhibition of L1 by *APOBEC3* occurs at the post-transcriptional level by a deamination-dependent or independent mechanism. Various studies suggest that representatives of the *APOBEC3* family of proteins have different mechanisms of inhibition of L1 [119,358,360]. The most active enzyme with respect to L1 hA3A has deaminase activity and converts cytosine to uracil in the first strand of the L1 cDNA transcript. As a result of such modification, the L1 copy undergoes further degradation [360,361]. A different mechanism has been identified for hA3C and hA3DE: acting by a deamination-independent mechanism, the enzyme blocks the L1 reverse transcription reaction by interacting with the L1 complex of RNP and ORF1 in the cell cytoplasm [362,363]. hA3B is less able to inhibit the reverse transcriptase activity of L1. It has an exclusively nuclear arrangement and appears to use an alternative mechanism [363,364,365]. In addition to the *APOBEC3* family, which is present only in humans, other types of proteins that are evolutionarily more conservative with deaminase activity are also active against L1: *AID* [366], *APOBEC1* [314], and ADAR1 and ADAR2 [367,368]. *APOBEC1* deaminase, similar to other enzymes, is active against viruses and retrotransposons. Interestingly, *APOBEC1* acts by a deamination-independent mechanism with respect to L1, while, with respect to other repeats and viruses, it uses a deamination-dependent mechanism [314]. *AID* [366] and *ADARs* [367,368] also act using the deamination-independent mechanism. Expression of *APOBEC3*, *AID*, and *APOBEC1* deaminases is observed in various somatic tissues and a high level of expression is observed in the connective tissues (blood and adipose tissue); in the organs of the immune, digestive, respiratory, and reproductive systems; and in embryonic stem cells (*A3B*, *A3C*, *A3D*, *A3F,* and *A3G*) [244,369]. All deaminases have a low level of expression in the brain, with the exception of *ADARs* which have a high level of expression in various parts of the brain [244].

#### 5.5.5. L1 Integration into the Genome and DNA Repair Mechanism

At the last stage of retrotransposition, which is the integration into the genome, the inhibitors are proteins involved in the repair of single and double-stranded DNA breaks (Figure 3 and Figure 5). It was initially revealed that double-stranded DNA breaks and defects in repair factors of non-homologous end-joining (NHEJ) protein kinase DNA-PKCs (PRKDC, XRCC7) and XRCC4 can contribute to L1 retrotransposition [49,50]. At the same time, some factors, such as DCLRE1C (artemis), LIG4, and XRCC6 (Ku70/Ku80), participate in retrotransposition and contribute to the truncation of L1 c 5′UTR, thus limiting the distribution of full-length active copies [46]. An effect on L1 activity was also detected for factors involved in the repair of single-stranded breaks using the nucleotide excision repair (*NER*) mechanism [123,124]. The dual role of the ERCC1/XPF complex in the regulation of L1 retrotransposition was previously suggested in one study [123]. The authors showed that the XPF factor inhibits L1 integration, while a deficiency of the ERCC1 factor leads to a decrease in L1 retrotranspositions [123]. Later, the details of the L1 regulation using *NER* factors were specified and a possible mechanism for inhibiting L1 retrotransposition was suggested [124]. Then, the XPA factor joins the ERCC1-XPF complex, which contributes to its enzymatic activity at the L1 integration site [370]. Interestingly, the lack of *NER* factors leads to an increase in the length of the targeted site duplication [124]. Defects in *NER* genes are associated with diseases of xeroderma pigmentosum (XP) and Cockayne syndrome [371,372].

A large contribution to the inhibition of L1 is made by factors involved in postreplicative DNA repair. One of the first discovered was the *ATM* gene, mutations in which lead to ataxia-telangiectasia [373]. In response to damage, *ATM* triggers the factors CHK2, p53, BRCA1, and the MRN complex (MRE11, Rad50, and NBS1), involved in the repair of post-replicative DNA and cell cycle regulation factors [373,374,375]. A lack of the *ATM* enzyme leads to an increase in L1 activity [45]. In response to cellular stress, repair factors are activated, combining various DNA repair pathways including *NER*, translesion synthesis, and homologous recombination [375]. An increase in L1 activity was detected in cases of deficiency of the DNA repair factor FANCD2 and SLX4, defects of which are associated with Fanconi Anemia (FA) disease [376]. The SLX4 complex blocks the distribution of L1 with the complex formed during the retrotransposition in the replication fork [377]. The factor FANCD2 is an activator and platform for the formation of the repair complex SLX4 for its aggregation with other factors including ERCC4, MUS81, and FAN1 [375]. Moreover, FANCD2 activates the translesion synthesis factor, RAD18 ubiquitin ligase, which is also involved in the inhibition of L1 activity. RAD18 causes monoubiquitinylation of the sliding clamp protein PCNA, which interacts with L1 ORF2 during retrotransposition [43,378]. Thus, RAD18 may possibly limit the insertion of L1 into the genome [377]. At the same time, the interaction of the Rad-6 domain (E2 ubiquitin-conjugated enzyme-binding domain) of the Rad18 factor with L1 ORF1 proteins was found, leading to the formation of P bodies and stress granules [377]. In addition, the Rad18 protein also suppresses Alu retrotransposons and HIV-1 DNA elements [377,379,380]. In a recent study, the role of post-replicative repair factors, homologous recombination, and BRCA1 ubiquitin ligase in suppressing L1 retrotranspositions were analyzed [126]. The authors proposed the following mechanism: BRCA1 prevents the insertion of L1 into the replication fork by initiating double-stranded cleavage, resection, and urgent protective coating of DNA ends with RPA proteins. As a result, this mechanism leads to the formation of a targeted site duplication. In addition, in the cytoplasm, BRCA1 can inhibit the translation of ORF2 and the formation of functional L1 RNP [126].

#### 5.5.6. Regulation of L1 by Cell Cycle Factors

Cell cycle factors are involved in the regulation of L1 activity (Figure 3 and Figure 5). However, their effect on L1 activity is ambiguous. The 5′UTR promoter of the L1 active and evolutionarily close families not older than 20 mya (L1Hs, L1PA2, and L1PA3) has the region of the transcription factor p53 binding site. In vitro experiments have shown that interaction of p53 with the L1 promoter increases the expression of these repeats [381]. In addition, experiments on model organisms of the fruit fly and zebrafish revealed a mechanism of L1 suppression induced by p53 via activation of the piRNA-signaling pathway in germ cells [382]. A correlation was also found between p53 and the repressing histone marker H3K9me3 in the L1 5′UTR in zebrafish embryos [382]. In addition to this, it has been shown that p53 inhibits L1 in cancer-derived and normal lung tissue from human cell cultures by binding to the 5′UTR and stimulating local deposition of repressive histone marks [383]. Additionally, inhibition of L1 has been shown to occur by other transcriptional factors regulating the cell cycle, namely p21 and p27 [321]. These factors do not affect the transcription of L1 but bind to the ORF2 protein, thus preventing the integration of L1 [321]. In turn, activation of p21 and p27 can occur using p53 and p73, for which L1 inhibition is also shown [321]. *MYC* proto-oncogene is involved in many cell processes and in the regulation of the cell cycle, the defects of which are often found in cancer [384]. *MYC* binding to the L1 5′UTR represses transcriptional activity. However, this has only been shown for some types of cancer cells [385]. Activity of Dual Specificity Phosphatase 1 (DUSP1) downregulate L1 in cancers cells [386]. DUSP1 is also known as mitogen-activated protein kinase (MAPK) phosphatase-1 (MKP-1), which is involved in the negative regulation of cellular proliferation and the suppression of inflammation [386].

#### 5.5.7. Positive Regulation of L1

In the region of the 5′UTR (+83 to +101) L1HS, there is a binding site for the transcription factor RUNX3 (runt-domain transcription factor). The binding of this factor increases the expression and retrotransposition of L1HS elements. In addition, another RUNX3 binding site is located on the antisense chain of the L1HS promoter (+526 to +508), thus expression also occurs in the antisense orientation of nearby sequences [387]. The transcription factor RUNX3 is expressed in many human tissues and the highest level of expression is found in the blood, skin, lungs, and digestive organs, but expression of the transcription factor is practically absent in the brain [244,387]. The L1 promoter region also contains two SRY-binding sites for the SOX transcription factor family [388], expressed in embryonic tissues, testes, and in various tissues in the adult body with the highest expression level in the brain, especially for the SOX2 factor [244]. SOX11 regulates L1 during the differentiation of neuronal tissues [388,389]. SOX2 enhances L1 expression in hippocampal neuronal stem cells in the adult brain [113,390]. Histone demethylase KDM4B is also a positive regulatory factor for L1, which promotes the demethylation of H3K9me3 histones [391]. Its increased activity is observed for various types of cancers [392,393,394]. The transcription factor FOXA1, the activity of which is associated with embryonic and postembryonic development, as well as with various cancers [395], can act as an activator of L1 expression [291]. In a recent study, its positive regulation of L1 was found during cellular ageing and senescence-associated secretory phenotypes (SASP), characterized by increased expression of immune factors [291,396]. In addition, in this phenotype, there is a decrease in the activity of the TREX1 and RB1 involved in the repression of L1, despite an increase in the level of interferon I [291]. The authors suggest that L1 activation and the inflammation caused by this activation are the hallmarks of ageing, and therefore a change in L1 regulation is a potential target for the treatment of age-related disorders. CTCF is involved in L1 activation, colocalizing with *MYC* in the L1 5′UTR promoter and 3′UTR region, and acts as an *MYC* repressor [385]. Moreover, CTCF also forms a complex with Cohesin subunit Rad21, for which there is also the evidence of positive regulation of L1 [397]. In the region of the L1 5′UTR transcript, there are binding sites of the internal ribosome entry site (IRESs), through which it binds to heterogeneous nuclear ribonucleoproteins and to the NCL nuclein, the latter being a positive factor for L1 activity [308]. Its interaction with L1 increases the level of expression of ORF1 (Figure 3 and Figure 5) [146,308,398]. It is also worth noting that in a recent study of cancer research, the ability to bind L1 was found for many transcription factors, among which new and previously described ones were found, such as ESR1, *MYC*, CCTCF, FOXA1, NR2F2 and E2F1, etc. Moreover, the authors showed the formation of new binding sites in the case of L1 truncation [399].

## 6. Factors Affecting Changes in L1 Regulation in Neuropsychiatric Diseases

Normally, L1 can be active in the brain [113,138,139,234,235,236]. In some neuropsychological pathologies, changes in L1 activity were detected. The most pronounced increase in L1 activity was found in Rett syndrome [241] and autism [400], as well as in ataxia telangiectasia [45]. The genetic causes that lead to an increase in L1 activity have been mostly studied with Rett syndrome and ataxia telangiectasia, and are associated with damage in the *MEPC2* and *ATM* genes [45,113]. The L1 control mechanism of these genes is described above. Some trends are observed in schizophrenia [401,402] and major depressive disorder [403]. However, the causes and factors that change the activity of L1 elements remain unknown for most diseases. Recently, some studies demonstrated the connection of genetic factors associated with neurodegenerative pathologies and L1 activity. One of these factors is the TAR DNA-binding protein (TDP-43), which is able to bind DNA and RNA, and is involved in the regulation of many processes [404]. TDP-43 is associated with neuropsychiatric pathologies such as amyotrophic lateral sclerosis (ALS) and frontotemporal degeneration (FTD) [405]. The protein cleavage, hyperphosphorylation, and aggregation in the form of ubiquitinated granules in the cytoplasm occur in the pathologies. Similar the TDP-43 “proteinopathy” occurs in other neurodegenerative diseases such as Alzheimer’s disease [406], Parkinson’s disease [407], and Huntington’s disease [408], and also with hereditary inclusion body myopathy (HIBM) [409]. Controversial results have been obtained regarding the effect of TDP-43 on L1 activity. TDP-43 is involved in many processes that can affect L1 activity, such as in autophagy, which contributes to the destruction of L1 stress granules [410], and in double-stranded DNA repair, wherein it binds to the damaged site and provides further formation of the XRCC4-DNA ligase IV complex, the activity of which can contribute to retrotransposition [49,50,411]. Additionally, in one of the latest studies, data were obtained regarding the inhibitory effect of TDP-43 on L1 activity and its absence was found to increase the level of L1 retrotranspositions by chromatin decompactivation [412]. Despite this, other studies obtained different results, showing that TDP-43 regulates the transcription of many genes and retrotransposons of Alu elements, and does not affect the activity of L1 elements [413,414]. In addition, an increase in HERV-K retroviral repeats was noted, while no changes in L1 activity were detected in lateral amyotrophic sclerosis [415,416]. The change in the expression of retrotransposons is also associated with the Tau protein encoded by the *MAPT* (microtubule-associated protein tau) gene [417,418]. Tau pathology is observed in various neurodegenerative disorders including Alzheimer’s disease [419,420]. The Tau protein hyperphosphorylates and forms hyperphosphorylated insoluble aggregates called neurofibrillary tangles [421,422,423]. One study showed activation of various retrotransposons, including L1 that lost the ability to retrotranspose due to accumulated mutations in the transcriptional reading frame [418]. We revaled no significant changes in the copy number of L1 in Alzheimer’s disease [136]. Another study showed an increase in the expression of endogenous retroviruses, but not of active L1, as a result of chromatin decondensation and a decrease in both piRNA and piwi proteins associated with Tau pathology in Alzheimer’s disease [417]. Mitochondrial dysfunction and oxidative stress are characteristic features of a number of diseases such as some forms of ataxia, neurodegenerative diseases (Parkinson’s disease in particular), various forms of cancer, and other diseases [424,425,426,427]. Recent studies have shown that abnormalities and a deficiency of both the mitochondrial chain and oxidative stress cause DNA hypomethylation and increased L1 activity [428,429,430]. The stress sensor *GABB45B* gene was connected to the death of dopaminergic neurons in Parkinson’s disease [431]. A recent study in mice shows that overexpression of Gadd45b leads to disorganized heterochromatin, increased DNA damage, vulnerability to oxidative stress, and further stable changes in DNA methylation, particularly in introns of neuronal genes harboring L1 [432].

## 7. Conclusions

The process of regulating the activity of L1 retrotransposons is a complex multifactor orchestra, the coordinated activity of which is extremely important for the healthy development of the organism. Factors that control L1 activity are involved in many biological processes, including cell division, immune response, ageing, and neuronal functions. Many cellular factors are aimed at limiting the activity and repropositions of L1 in genome. A much smaller number of factors positively regulate L1. L1 activity is necessary in some stages of development. The functional role of L1 elemets is intriguing and not yet fully elucidated. Recent studies showed the L1 contribution to the early embryonic development, the inflammation, the cell cycle and cellular senescence. Recently, a major breakthrough has been made in understanding the molecular genetic mechanisms of the retrotransposition and regulation of L1 activity. However, many factors involved in the process of the retrotransposition and regulation of L1 in pathologies are not yet known.

Moreover, this research area also has prospects for understanding the pathogenesis and development of therapies for autoimmune and viral diseases, neuropsychiatric disorders, and oncological diseases. The strict regulation of L1 elements during germline and embryonic development, as well as the various defects in L1 regulatory factors have been extensively studied on mouse models. Thus, this provides a foundation for research in human reproduction and embryology. The investigation of genetic factors that regulate L1 activity and their defects is also of great importance in the research of rare and complex diseases and, potentially, modulation of certain disease conditions vis regulation of the retrotransposons.

## Data Availability

Not applicable.
